# Optical Coherence Tomography Noise Reduction Using Anisotropic Local Bivariate Gaussian Mixture Prior in 3D Complex Wavelet Domain

**DOI:** 10.1155/2013/417491

**Published:** 2013-10-10

**Authors:** Hossein Rabbani, Milan Sonka, Michael D. Abramoff

**Affiliations:** ^1^Biomedical Engineering Department, Medical Image & Signal Processing Research Center, Isfahan University of Medical Sciences, Isfahan 81745, Iran; ^2^The Iowa Institute for Biomedical Imaging, The University of Iowa, Iowa City, IA 52242, USA

## Abstract

In this paper, MMSE estimator is employed for noise-free 3D OCT data recovery in 3D complex wavelet domain. Since the proposed distribution for noise-free data plays a key role in the performance of MMSE estimator, a priori distribution for the pdf of noise-free 3D complex wavelet coefficients is proposed which is able to model the main statistical properties of wavelets. We model the coefficients with a mixture of two bivariate Gaussian pdfs with local parameters which are able to capture the heavy-tailed property and inter- and intrascale dependencies of coefficients. In addition, based on the special structure of OCT images, we use an anisotropic windowing procedure for local parameters estimation that results in visual quality improvement. On this base, several OCT despeckling algorithms are obtained based on using Gaussian/two-sided Rayleigh noise distribution and homomorphic/nonhomomorphic model. In order to evaluate the performance of the proposed algorithm, we use 156 selected ROIs from 650 × 512 × 128 OCT dataset in the presence of wet AMD pathology. Our simulations show that the best MMSE estimator using local bivariate mixture prior is for the nonhomomorphic model in the presence of Gaussian noise which results in an improvement of 7.8 ± 1.7 in CNR.

## 1. Introduction 

Optical coherence tomography (OCT) is an optical signal acquisition and processing method that captures 3D images from within optical scattering media such as biological tissues [1–4]. For example, in ophthalmology, OCT is used to obtain detailed images from within the retina [4]. Similar to other optical tomographic techniques, OCT suffers from speckle noise that reduces the ability of image interpretation [5]. So, noise reduction is an essential part of OCT image processing systems. Until now, several techniques for OCT noise reduction have been reported [6–14]. Initial methods perform in complex domain [15], that is, before producing magnitude of OCT interference signal, while most introduced despeckling methods are applied after an OCT image is formed [6–14]. These methods, which usually suppose multiplicative noise for speckled data, also can be categorized into image domain and transform domain methods. As an example for image domain techniques, in [16] the rotating kernel transform (RKT) filters are applied on an image with a set of oriented kernels and keep the largest filter output for each pixel. Other image domain methods based on enhanced Lee filter [17], median filter [17], symmetric nearest neighbor filter [17], and adaptive Wiener filter [17], and I-divergence regularization [6] and PDE-based nonlinear diffusion methods [14] have been reported in the literature.

Transform domain techniques typically outperform the image domain techniques because incorporating speckle statistics in the despeckling process would be facilitated in sparse domains. Such techniques apply a sparse transform (such as wavelet and curvelet transforms) [7–12, 18] directly on data (viz., nonhomomorphic methods) or on log-transformed data (viz., homomorphic methods), and suppose that in the sparse domain noise is converted to additive white Gaussian noise (AWGN) [13] or other models which can be removed using an appropriate shrinkage function. For example, in [18], a spatially adaptive wavelet thresholding method is used for speckle suppression in log-transformed domain. Since actual signal in OCT images consists of horizontal edges arising from reflections at the layer boundaries, most of the edge information is in “low-pass”-“high-pass” (LH) subbands (and some of it in HH subbands). Therefore, an increased threshold in the vertical subbands using a constant multiplier (*K* = 4) is chosen to decrease further noise with a minimal effect on the edge sharpness. Other transform domain methods based on hard thresholding in 3D curvelet domain [8], soft thresholding in discrete complex wavelet transform (DCWT) domain [9], and temporal and spatial wavelet-based filtering [10] have been reported in other literatures.

In fact denoising is the problem of obtaining the noise-free data from noisy data observation, which may be solved in a deterministic or probabilistic framework. In the first case, each voxel is considered as an unknown deterministic variable, and non-Bayesian techniques are employed to solve this problem. In the second case, the data is modeled as a random field, and Bayesian methods are used for the estimation of clean data from the noisy environment. Therefore, the proposed prior probability distributions for noise-free data and noise (i.e., proposed as speckle for OCT data) play a key role in the noise reduction problem.

### 1.1. Statistical Properties of Noise-Free Coefficients

Description of the statistical properties of natural signals can be facilitated in the wavelet domain [19] due to sparseness and decorrelation properties of wavelets [20]. The sparseness property states that the marginal pdf of wavelet coefficients in each subband has a large peak at zero and its tails fall to zero slower than the Gaussian pdf (leptokurtic). On this base, some long-tailed pdfs such as generalized Gaussian distribution (GGD) [21, 22], *α*-stable distributions [23], Bessel K form densities [24, 25], and mixture pdfs [26–31] have been proposed. Although the decorrelation property of wavelets states that coefficients at the same positions in the adjacent scales are uncorrelated, it does not mean that they are independent. The interscale dependency of wavelet states that large/small values of wavelet coefficients tend to propagate across scales [32]. Some researchers have proposed hidden Markov models (HMMs) [33] and Markov random fields (MRFs) [34] to model the interscale dependency [35]. Recently, it has been shown that some non-Gaussian bivariate joint pdfs for each coefficient and its parent, such as circular symmetric Laplacian pdf [36], bivariate Cauchy distribution [37], (multivariate) Gaussian scale mixture (GSM) model [27, 38, 39], and bivariate Laplacian mixture models [40] are able to capture this property easily and produce better denoising results with lower computational complexity.

The dependencies between wavelet coefficients are not restricted to the interscale dependency. There is another dependency between spatial adjacent coefficients in each subband, namely, intrascale dependency [42]. This dependency states that if a particular wavelet coefficient is large/small, then the spatial adjacent coefficients are likely to be large/small too. Usually this property is captured using local parameters for pdfs [37], and it has been shown that denoising algorithms using this property for statistical modeling of wavelets are able to improve the denoising results [43–45]. For example, Mihçak [43] employs local variance for Gaussian pdf to model intrascale dependency. In [44], a mixture of two Laplace pdf with local parameters is proposed for simultaneously capturing heavy-tailed nature and intrascale dependency. Reference [45], using local variance for proposed model in [36], improves the results for noise reduction application because this local pdf models both interscale and intrascale dependencies. In this paper, we extend the proposed pdf in [46] based on a mixture of bivariate Gaussian pdfs with local parameters for noise-free wavelet coefficients. Since the empirically observed distribution of wavelet coefficient pairs in adjacent scales have elliptical symmetry, we use different variances for marginal pdfs that lead to an elliptical symmetric bivariate pdf instead of circular symmetric pdf. Recently, it has been shown that using anisotropic window instead of square window can improve the denoising results [47]. Based on the special structure of OCT data, we choose an anisotropic windowing procedure for local parameters estimation that results in visual quality improvement.

### 1.2. Discrete Complex Wavelet Transform (DCWT)

The wavelet based image denoising consists of the following steps.

1. Signal transformation of the noisy observation.
2. Modification of the noisy wavelet coefficients based on some criteria.
3. Inverse signal transformation of modified coefficients.

As explained earlier, the second step depends on the type of estimator and for a minimum mean square error (MMSE) estimator, the proposed model for signal and noise (which we propose as a multiplicative model), the proposed pdf of noise-free wavelet coefficients (modeled, in this paper, as a mixture of bivariate Gaussian pdfs with local parameters), and the proposed pdf for noise (with which we test both Gaussian and two-sided Rayleigh distributions) define the performance of the algorithm. However, for the first and last steps of wavelet-based denoising algorithm, the type of transformation plays a key role. In this paper, we use DCWT [48] instead of ordinary discrete wavelet transform (DWT). Despite DWT being a sparse representation that outperforms many signal processing approaches, it does not lead to an optimum performance in all applications and suffers from several fundamental shortcomings (especially in high-dimensional cases), which DCWT avoids them. These shortcomings are as follows.

**Figure 1:**
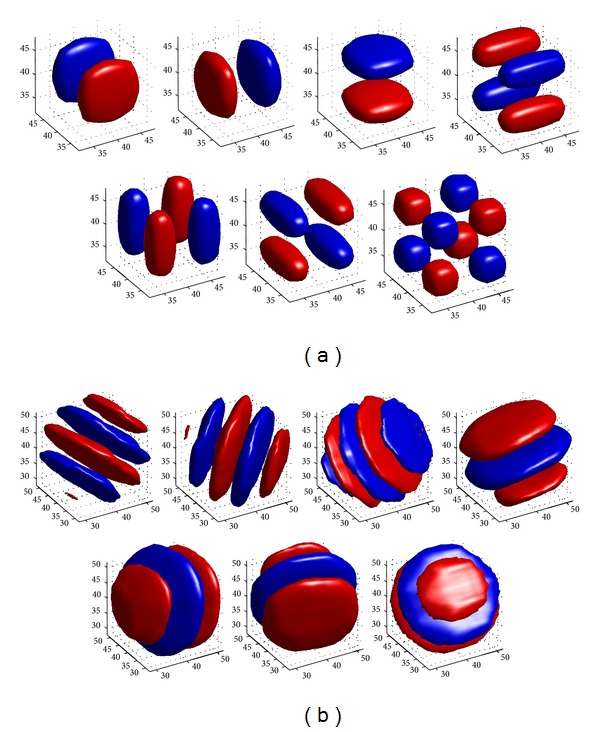
A comparison between the idealized support of the Fourier spectrum of each standard and complex wavelet in the 3D frequency domain. (a) Isosurfaces of the 7 3D wavelets for a standard 3D wavelet transform. The blue and red colors have the same amplitude, but their phases are complement. (b) Isosurfaces of 7 of the 28 3D wavelets for a 3D DCWT. Each subband corresponds to motion in a specific direction [41].

1. In the neighborhood of an edge, the DWT produces both large and small wavelet coefficients. In contrast, the magnitudes of DCWT coefficients are more directly related to their adjacency to the edge. The main reason of this phenomenon is using bandpass filters that produce DWT coefficients which oscillate positively and negatively around the singularities, and this subject complicates wavelet-based processing.
2. DWT is not shift invariant. It means that a small shift in the input signal of DWT makes the total energy of wavelet coefficients in subband completely differ. This shift greatly perturbs oscillation pattern around singularities of the DWT coefficient which complicates wavelet-domain processing.
3. Since the DWT coefficients in each subband are produced via critical sampling after using nonideal low-pass and high-pass filters, substantial aliasing would be produced. If the wavelet coefficients are not changed, the inverse DWT cancels this aliasing. Applying any processing method on wavelet coefficients (such as thresholding) disarranges this balance between the forward and inverse transforms which leads to artifacts in the reconstructed signal.
4. The directional selectivity of 2D DCWT has been explained in Appendix A. Similar to the 2D case, the standard 3D data transforms, which are separable multiplication of 1D tensors, do not provide useful representations with good energy compaction property for 3D data. For example, the multi-dimensional standard separable DWT mixes orientations and motions in its subbands and produces the checkerboard artifacts (Figure 1). In contrast, since the spectrum of the (approximately) analytic 1D wavelet is supported on only one side of the frequency axis, the spectrum of the DCWT in 3D domain is supported in only 1/27 of the 3D frequency plane. So, instead of 3D DWT, usually oriented transforms such as 3D DCWT are proposed for 3D data processing [41, 48–52]. Figure 1 shows a comparison between subbands of 3D DWT and 3D DCWT.

### 1.3. Organization of the Paper

In Section 2, we explain our proposed pdf for noise-free 3D DCWT coefficients, that is, a mixture of bivariate Gaussian distributions with local parameters. In Section 3, at first we obtain a local thresholding function supposing a priori distribution as a bivariate Gaussian pdf with local variance, and then in a Bayesian framework we produce our new shrinkage functions derived from the proposed pdf and using Gaussian/two-sided Rayleigh noise distribution and homomorphic/non-homomorphic model. In Section 4, we explain the proposed anisotropic window selection procedure for local parameter estimation based on special structure of OCT data. In Section 5, we use our model for wavelet-based denoising of several 3D OCT data. We compare our methods visually and in terms of PSNR. Also in this section, we use the proposed method for nonspeckle noise reduction. Finally, in Section 6, we summarize this paper and suggest some future work.

## 2. Bivariate Gaussian Mixture Model with Local Parameters

**Figure 2:**
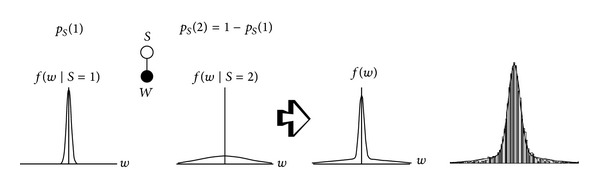
Zero-mean Gaussian mixture model (left image) and empirical histogram of wavelet in a subband together with the Gaussian mixture model (right image) [26].

**Figure 3:**
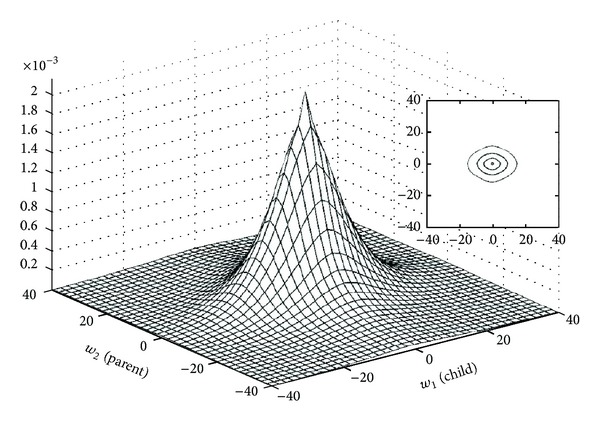
Empirical joint parent-child histogram of wavelet coefficients (computed from the Corel image database) [42].

One of the primary properties of the wavelet transform is compression. This property means that the marginal distributions of wavelet coefficients are highly kurtotic, and so long-tailed distributions are suitable models for marginal pdf. A zero-mean mixture model could have a large peak at zero and would be long tailed. For example, in [22, 26, 29, 31] a mixture of Gaussian distributions is proposed to model the heavy-tailed nature of wavelet coefficients.  Figure 2 shows this model that consists of two zero-mean Gaussian distributions with two different variances. The Gaussian pdf with low variance can model the large peak at zero and the Gaussian pdf with high variance can model tails of distribution. The secondary properties of the wavelet transform are clustering and persistence. The clustering property, that is called intrascale dependency, states that if a particular wavelet coefficient is large/small, then adjacent coefficients are very likely to also be large/small [36], and usually local pdfs are able to model this property. The persistence property, that is called the interscale dependency, states that large/small values of wavelet coefficients tend to propagate across scales [36]. As an example, Figure 3 illustrates the empirical joint parent-child histogram of wavelet coefficients computed from the 200, 512 × 512 images from the Corel image database [42]. Usually this property can be modeled using proper bivariate pdfs.

### 2.1. Description of the Proposed Model

In this paper, we assume a pdf as a mixture of two bivariate Gaussian pdfs with local parameters in order to model the distribution of wavelet coefficients of images as follows:

(1) pw−(k)(w−(k))=a(k)p1(w−(k))+(1−a(k))p2(w−(k))=a(k)e(w12(k)/2σ112(k))−(w22(k)/2σ122(k))2πσ11(k)σ12(k) +(1−a(k))e(−w12(k)/2σ212(k))−(w22(k)/2σ222(k))2πσ21(k)σ22(k),

where *a*(*k*) ∈ [0,1],  *σ*_11_(*k*),  *σ*_12_(*k*),  *σ*_21_(*k*),  *σ*_22_(*k*) are the mixture model parameters. For each random bivariable, the second component is the parent of the first component; for example, *w*_2_(*k*) represent, the parent of *w*_1_(*k*) at the same spatial position as the *k*th wavelet coefficient *w*_1_(*k*) and at the next coarser scale.

Our proposed model in this paper, that is a mixture of bivariate Gaussian pdfs with local parameters, is mixture, bivariate and local. Therefore, it is able to simultaneously capture the heavy-tailed property and inter- and intrascale dependencies.

After substitution of mixture model in the definition of *E*(*w*_1_(*k*)*w*_2_(*k*)), we can see that this pdf represents two uncorrelated random variables as follows:

(2) E(w1(k)w2(k))  =∬w1(k)w2(k)pw−(k)(w−(k))dw−(k)  =(1−a(k))   ×∬w1(k)w2(k)p2(w−(k))dw1(k)dw2(k)   +a(k)∬w1(k)w2(k)p1(w−(k))dw1(k)dw2(k)  =0.

Interestingly, the marginal pdf of *w*_*i*_(*k*) for *i* = 1, 2 is the mixture of two univariate Gaussian pdf with local parameters [44],

(3) pw1(k)(w1(k))=∫−∞∞pw−(k)(w−(k))dw2(k)=a(k)exp⁡(−w12(k)/2σ112(k))σ11(k)2π +(1−a(k))exp⁡(−w12(k)/2σ212(k))σ21(k)2π.

It is easy to see that *w*_1_(*k*), *w*_2_(*k*) are not independent; that is,

(4) $$\begin{array}{c} p_{\overset{-}{w}(k)}\left(\overset{-}{w}\left(k\right)\right)\neq p_{w_{1}(k)}\left(w_{1}\left(k\right)\right)p_{w_{2}(k)}\left(w_{2}\left(k\right)\right). \end{array}$$

See Appendix B for more explanation.

### 2.2. Local EM Algorithm

To characterize the parameters in (1), it is necessary to have the parameters *σ*_11_(*k*),  *σ*_21_(*k*), *σ*_12_(*k*), *σ*_22_(*k*), and *a*(*k*). For this mixture model, we use an iterative numerical algorithm to estimate these parameters. The expectation maximization (EM) algorithm is most frequently used to estimate such parameters. Usually, the EM algorithm for mixture models employs all data in each subband to obtain the parameters. Using this global EM algorithm, equal parameters are obtained for all data in each subband. However, to model the intrascale dependency, we must incorporate the local statistics and need to have different parameters for each voxel in each subband. So, we introduce a local version of EM algorithm. This local EM algorithm is able to obtain separate parameters for each voxel by the implementation of EM algorithm in each window *N*(*k*) centered at *w*(*k*). This iterative algorithm has two steps. Assuming that the observed data $\overset{-}{w}(k)$ for *k* = 1,…, *N*, the *E*-step calculates the responsibility factors for each data as follows:

(5) $$\begin{array}{c} r_{1}\left(k\right)⟵\frac{a\left(k\right)p_{1}\left(\overset{-}{w}\left(k\right)\right)}{a\left(k\right)p_{1}\left(\overset{-}{w}\left(k\right)\right)+\left(1-a\left(k\right)\right)p_{2}\left(\overset{-}{w}\left(k\right)\right)},\\ r_{2}\left(k\right)⟵1-r_{1}\left(k\right). \end{array}$$

The *M*-step updates the parameters *a*(*k*),  *σ*_11_(*k*),  *σ*_12_(*k*),  *σ*_21_(*k*),  and  *σ*_22_(*k*). *a*(*k*) is computed by

(6) $$\begin{array}{c} a\left(k\right)⟵\frac{1}{M}\underset{j\in N\left(K\right)}{\sum }r_{1}\left(j\right), \end{array}$$

where *M* is the number of coefficients in the square window *N*(*k*) centered at *w*(*k*). 

The variances *σ*_11_(*k*),  *σ*_12_(*k*),  *σ*_21_(*k*),  and  *σ*_22_(*k*) are computed by [40]:

(7) $$\begin{array}{c} \sigma_{im}^{2}\left(k\right)⟵\frac{\sum_{j\in N\left(K\right)}^{}r_{i}\left(j\right)w_{m}^{2}\left(k\right)}{\sum_{j\in N\left(K\right)}^{}r_{i}\left(j\right)},\;i,m=1,2. \end{array}$$

## 3. Denoising Using MMSE Estimator

In this section, the denoising of a 3D OCT data is considered. We assume that dominant noise in OCT data is speckle. In this case as a common model, we propose multiplicative model as follows:

(8) $$\begin{array}{c} x\left(i\right)=s\left(i\right)g\left(i\right), \end{array}$$

where *i* is the index of voxel and is between 1 and number of voxels. 

As explained in Introduction, reported transform-based OCT noise reduction methods in the literatures [7–12, 18] usually at first transform data into log domain, and suppose that noise in log domain is AWGN:

(9) $$\begin{array}{c} W\left(\log x\left(i\right)\right)=W\left(\log s\left(i\right)\right)+W\left(\log g\left(i\right)\right), \end{array}$$

where in this paper *W* shows 3D DCWT operator. So, we can write

(10) $$\begin{array}{c} y\left(k\right)=w\left(k\right)+n\left(k\right), \end{array}$$

where *w*(*k*), *y*(*k*), and *n*(*k*) are, respectively, the *k*th noise-free 3D DCWT coefficients, noisy 3D DCWT coefficients, and noise in the 3D DCWT domain.

Recently, it has been reported [56–59] that non-homomorphic techniques that do not use this nonlinear operation and apply wavelet transform directly on speckled data lead to unbiased estimation of the data and decrease the computational complexity. On this base after applying 3D DCWT (directly) on data, we would have

(11) W(x(i))=W(s(i)g(i))=W(s(i)+s(i)(g(i)−1))=W(s(i))+W(s(i)(g(i)−1)).

Again we can write

(12) $$\begin{array}{c} y\left(k\right)=w\left(k\right)+n\left(k\right), \end{array}$$

where *w*(*k*),  *y*(*k*), and *n*(*k*) are, respectively, the *k*th noise-free 3D DCWT coefficients, noisy 3D DCWT coefficients, and noise in the 3D DCWT domain. Since speckle noise *g* can be modeled as a unit-mean random process independent of the noise-free data, we would have *E*[*W*(*s*(*g* − 1))] = 0, and also it can be easily shown [58] that *E*[*W*(*s*)*W*(*s*(*g* − 1))] = 0 which means that *w*(*k*) and *n*(*k*) are zero-mean uncorrelated random variables. If *w*_*p*_(*k*), *y*_*p*_(*k*), and *n*_*p*_(*k*) show the parent coefficients of *w*(*k*), *y*(*k*), and *n*(*k*), respectively, we can write

(13) $$\begin{array}{c} y_{p}\left(k\right)=w_{p}\left(k\right)+n_{p}\left(k\right). \end{array}$$

Based on the persistence property, we need to have a bivariate model based on parent-child pairs. So, we can propose the following bivariate model:

(14) $$\begin{array}{c} \overset{-}{y}\left(k\right)=\overset{-}{w}\left(k\right)+\overset{-}{n}\left(k\right), \end{array}$$

where $\overset{-}{w}(k)=(w(k),w_{p}(k))$, $\overset{-}{y}(k)=(y(k),y_{p}(k))$, and $\overset{-}{n}(k)=(n(k),n_{p}(k))$ are, respectively, the *k*th parent-child pairs of noise-free 3D DCWT coefficients, noisy 3D DCWT coefficients, and additive noise in the 3D DCWT domain. In the literature, several models such as *K*-distribution, Rayleigh, Weibull, log-normal, and Nakagami distributions have been proposed [57, 58, 60–63] for speckle in image domain. In this paper, we test both AWGN and two-sided Rayleigh model for noise in wavelet domain as follows:

(15) $$\begin{array}{c} p_{\overset{-}{n}}\left(\overset{-}{n}\left(k\right)\right)=\frac{1}{2\pi \sigma_{n}^{2}}\exp\left(-\frac{n_{1}^{2}\left(k\right)+n_{2}^{2}\left(k\right)}{2\sigma_{n}^{2}}\right), \end{array}$$

(16) $$\begin{array}{c} p_{\overset{-}{n}}\left(\overset{-}{n}\left(k\right)\right)=\frac{\left|n_{1}\left(k\right)n_{2}\left(k\right)\right|}{4\alpha^{4}}\exp\left(-\frac{n_{1}^{2}\left(k\right)+n_{2}^{2}\left(k\right)}{2\alpha^{2}}\right), \end{array}$$

where *σ*_*n*_^2^ = 2*α* ^2^ shows the noise variance. 

Now our goal is the estimation of $\overset{-}{w}(k)$ from $\overset{-}{y}(k)=\overset{-}{w}(k)+\overset{-}{n}(k)$, where $\overset{-}{n}(k)$ is a Gaussian or two-sided Rayleigh according to some criteria.

If we employ the MMSE estimator for the estimation problem, we get the posterior mean as an optimal solution:

(17) w^(k)=∬w(k)pw−(k) ∣ y−(k)(w−(k) ∣ y−(k))dw−(k)=∬w(k)py−(k) ∣ w−(k)(y−(k) ∣ w−(k))pw−(k)(w−(k))py−(k)(y−(k))dw−(k)=∬w(k)py−(k) ∣ w−(k)(y−(k) ∣ w−(k))pw−(k)(w−(k))dw−(k)py−(k)(y−(k))=∬w(k)py−(k) ∣ w−(k)(y−(k) ∣ w−(k))pw−(k)(w−(k))dw−(k)∬py−(k) ∣ w−(k)(y−(k) ∣ w−(k))pw−(k)(w−(k))dw−(k)=∬w(k)pn−(y−(k)−w−(k))pw−(k)(w−(k))dw−(k)∬pn−(y−(k)−w−(k))pw−(k)(w−(k))dw−(k).

### 3.1. Denoising Based on Modeling Noise-Free Data by Bivariate Gaussian PDF with Local Variance

In order to solve (17), we must know the prior distribution of 3D DCWT coefficients, that is, $p_{\overset{-}{w}(k)}(\overset{-}{w}(k))$. Defining $\text{Gauss}(x,\sigma ):=\exp(-x^{2}/(2\sigma^{2}))/(\sigma \sqrt{2\pi })$, if we suppose that *w*(*k*), *w*_*p*_(*k*) are independent Gaussian pdf with variances *σ*_1_(*k*) and *σ*_2_(*k*), the following bivariate Gaussian pdf with local variances can be proposed for the noise-free wavelet coefficients:

(18) pw−(k)(w−(k))=pw(k)(w(k))·pwp(k)(wp(k))=Gauss(w(k),σ(k))·Gauss(wp(k),σp(k))⇒pw−(k)(w−(k))=exp⁡[−w2(k)2σ2(k)−wp2(k)2σp2(k)] ×(2πσ(k)σp(k))−1.

In this case, *w*(*k*) and *w*_*p*_(*k*) are uncorrelated and independent, and therefore the MMSE estimator of *w*(*k*), *w*_*p*_(*k*) yields the shrinkage function corresponding to univariate Gaussian pdf, that is, Wiener filter [21] as follows:

(19) w^(k)=∬w(k)pn−(y−(k)−w−(k))pw−(k)(w−(k))dw−(k)∬pn−(y−(k)−w−(k))pw−(k)(w−(k))dw−(k)=y(k)σ2(k)σ2(k)+σn2.

And so we can write

(20) $$\begin{array}{c} \hat{\overset{-}{w}}\left(k\right)=\left(\frac{y\left(k\right)\sigma^{2}\left(k\right)}{\sigma^{2}\left(k\right)+\sigma_{n}^{2}},\;\;\frac{y_{p}\left(k\right)\sigma_{p}^{2}\left(k\right)}{\sigma_{p}^{2}\left(k\right)+\sigma_{n}^{2}}\right). \end{array}$$

Similarly, if we choose two-sided Rayleigh pdf for noise distribution, the following estimator is obtained [44]:

(21) w^(k)=2z(k)2(2−σ2(k)α2)+π2(1−2σ2(k)z2(k)α2) ×(erfc⁡x(z(k))−erfc⁡x(−z(k))) ×(1α2+1σ2(k)(2+z(k)πerfc⁡x(−z(k))   −z(k)πerfc⁡x(z(k))))−1,

where

(22) $$\begin{array}{c} z\left(k\right)=\frac{y\left(k\right)}{\sigma^{2}\left(k\right)}\sqrt{\frac{1}{2/\alpha^{2}+2/\sigma^{2}\left(k\right)}},\\ \mathrm{erfc}x\left(u\right)=\frac{2}{\sqrt{\pi }}\overset{\infty }{\underset{0}{\int }}e^{-t^{2}-2tu}dt. \end{array}$$

And so we can write

(23) w−^(k)=((2z(k)2(2−σ2(k)α2) +π2(1−σ2(k)z2(k)α2) ×(erfc⁡x(z(k))−erfc⁡x(−z(k)))) ×(1α2+1σ2(k) ×(2+z(k)πerfc⁡x(−z(k))   −z(k)πerfc⁡x(z(k))))−1, (2z(k)2(2−σp2(k)α2)+π2(1−σp2(k)z2(k)α2)×(erfc⁡x(zp(k))−erfc⁡x(−zp(k)))) ×(1α2+1σp2(k)×(2+zp(k)πerfc⁡x(−zp(k))  −zp(k)πerfc⁡x(zp(k))))−1).

Suppose that the input noise variance is known. To implement (20) or (23), we must know the parameter of the prior *σ*(*k*) (suppose that *σ*(*k*) = *σ*_*p*_(*k*)). Mihçak et al. [43] showed that using local variance (instead of global variance) for Wiener filter leads to a substantial improvement in denoising results (using local variance allows incorporating the local statistics of image into the proposed prior). It has been shown in the literature that the correctness of estimation of variance is an impact factor for denoising [23, 27, 34, 42–46]. Thus, the proposed criteria for estimation of the variance, such as the involved data for estimation (e.g., in some approaches the coarser scales are used as a source of prior), the type of estimator, and the shape and size of the proposed window for the local estimation of the variance, play key roles in the performance of denoising procedure. For example, in [54] a recurrence equation using a local Gaussian pdf is used for estimation of *σ*(*k*) or in [64] the variable size of the locally adaptive window is obtained using a region-based approach. However, for each data point $\overset{-}{y}(k)$, a simple estimation of *σ*(*k*) can be formed based on a local neighborhood *N*(*k*). In simplest case, we can use a square window *N*(*k*) centered at $\overset{-}{y}(k)$ and suppose that in this window the variance is approximately constant. Then, an empirical estimate for *σ*(*k*) can be obtained as follows:

(24) $$\begin{array}{c} {\hat{\sigma }}^{2}\left(k\right)=\frac{1}{2M}\underset{j\in N\left(k\right)}{\sum }\left(y^{2}\left(j\right)+y_{p}^{2}\left(j\right)\right)-\sigma_{n}^{2}, \end{array}$$

where *M* is the number of coefficients in *N*(*k*) and *σ*_*n*_ can be estimated by [4] *σ*_*n*_ = median{*|*noisy wavelet coefficients in finest scale*|*}/0.6745. In this estimation, we propose the coarser scale as a source of prior, but another estimate can be obtained using only spatial adjacent in the same scale. It has been shown [47] that the local features in the edges of images are not isotropic and so can be better modeled in a shape-adaptive window selection manner. We explain in this regard in Section 4 and try to improve the denoising results by using anisotropic window instead of square window for the estimation of local parameters (such as variance in (24)).

### 3.2. Denoising Based on Modeling Noise-Free Data by a Mixture of Bivariate Gaussian PDFs with Local Parameters

A nonlinear shrinkage function for wavelet-based denoising, which is derived by assuming that the noise-free wavelet coefficients follow a bivariate Gaussian mixture model with local parameters given by (1), is introduced in this section. Substituting (1) in (17), we can write

(25) w^(k)=(∬w(k)pn−(y−(k)−w−(k))×[a(k)p1(w−(k))+(1−a(k))p2(w−(k))]dw−(k)) ×(∬pn−(y−(k)−w−(k))×[a(k)p1(w−(k))+(1−a(k))p2(w−(k))]dw−(k))−1=a(k)∬w(k)pn−(y−(k)−w−(k))p1(w−(k))dw−(k)a(k)g1(y−(k))+(1−a(k))g2(y−(k)) +((1−a(k))×∬w(k)pn−(y−(k)−w−(k))p2(w−(k))dw−(k)) ×(a(k)g1(y−(k))+(1−a(k))g2(y−(k)))−1,

where

(26) gi(y−(k))=∬pn−(y−(k)−w−(k))pi(w−(k))dw−(k),i=1,2.

In fact, $g_{i}(\overset{-}{y}(k))$ is the 2D convolution of the pdf of $p_{\overset{-}{n}}$ (defined in (15) or (16)) and *p*_*i*_ (defined in (1)). Using (15) as proposed model for noise, both $p_{\overset{-}{n}}$ and *p*_*i*_ are bivariate Gaussian pdfs. So, we obtain

(27) gi(y−(k))=exp⁡(−(1/2)×(y2(k)/(σn2+σi12(k))+yp2(k)/(σn2+σi22(k)))) ×(2π(σn2+σi12(k))(σn2+σi22(k)))−1i=1,2.

For two-sided Rayleigh noise (16), more computations are needed. After some simplifications, the final formula would be

(28) gi(y−(k))  =exp⁡(−y2(k)/2σi12(k)−yp2(k)/2σi22(k))8π(1+σi12(k)/α2)(1+σi22(k)/α2)σi1(k)σi2(k)   ×(2+zi(k)πerfc⁡x(−zi(k))  −zi(k)πerfc⁡x(zi(k)))   ×(2+zip(k)πerfc⁡x(−zip(k))  −zip(k)πerfc⁡x(zip(k))),              i=1,2,

where

(29) $$\begin{array}{c} z_{i}\left(k\right)=\frac{y\left(k\right)}{\sigma_{i1}^{2}\left(k\right)}\sqrt{\frac{1}{2/\alpha^{2}+\left(2/\sigma {}_{i1}^{2}\left(k\right)\right)}},\;i=1,2,\\ z_{ip}\left(k\right)=\frac{y_{p}\left(k\right)}{\sigma_{i2}^{2}\left(k\right)}\sqrt{\frac{1}{2/\alpha^{2}+\left(2/\sigma_{i2}^{2}\left(k\right)\right)}},\;i=1,2. \end{array}$$

Using (19), we can obtain numerators of (25), and finally (25) for AWGN can be written as

(30) w^(k)=(σ112(k)/(σ112(k)+σn2)+R(y−(k))(σ212(k)/(σ212(k)+σn2))) ×(1+R(y−(k)))−1y(k),

where

(31) R(y−(k))=(((1−a(k)) ×exp⁡(−12 ×(y2(k)σn2+σ212(k)+yp2(k)σn2+σ222(k)))) ×((σn2+σ212(k))(σn2+σ222(k)))−1) ×((a(k)exp⁡(−12(y2(k)σn2+σ112(k)+yp2(k)σn2+σ122(k))))  ×((σn2+σ112(k))(σn2+σ122(k)))−1)−1.

**Figure 4:**
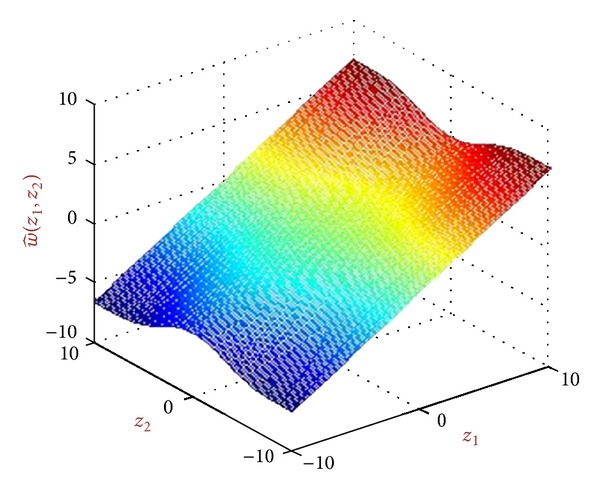
A shrinkage function produced from BiGaussMixShrink for sample parameters.

We call the new obtained bivariate local shrinkage function as *BiGaussMixShrinkL*.  Figure 4 shows this shrinkage function with sample constant parameters.

Similarly, using (21), we can obtain numerators of (25), and finally (25) for two-sided Rayleigh noise is obtained as

(32) w^(k)=11+R(y−(k)) ×(2z1(k)2(2−σ112(k)α2)+π2(1−σ112(k)z12(k)α2)×(erfc⁡x(z1(k))−erfc⁡x(−z1(k))))×(1α2+1σ112(k)×(2+z1(k)πerfc⁡x(−z1(k))  −z1(k)πerfc⁡x(z1(k))))−1 +R(y−(k))1+R(y−(k)) ×(2z2(k)2(2−σ212(k)α2)+π2(1−σ212(k)z22(k)α2)×(erfc⁡x(z2(k))−erfc⁡x(−z2(k))))×(1α2+1σ212(k)×(2+z2(k)πerfc⁡x(−z2(k)) −z2(k)πerfc⁡x(z2(k))))−1,

where

(33) R(y−(k))  =1−a(k)a(k)   ×(1+σ112(k)/α2)(1+σ122(k)/α2)σ11(k)σ12(k)(1+σ212(k)/α2)(1+σ222(k)/α2)σ2i1(k)σ22(k)   ×exp⁡⁡(−y2(k)/2σ212(k)−yp2(k)/2σ222(k))exp⁡⁡(−y2(k)/2σ112(k)−yp2(k)/2σ122(k))   ×((2+z2(k)πerfc⁡x(−z2(k))−z2(k)πerfc⁡x(z2(k)))   ×(2+z2p(k)πerfc⁡x(−z2p(k)) −z2p(k)πerfc⁡x(z2p(k))))   ×((2+z1(k)πerfc⁡x(−z1(k))−z1(k)πerfc⁡x(z1(k)))   ×(2+z1p(k)πerfc⁡x(−z1p(k))  −z1p(k)πerfc⁡x(z1p(k))))−1.

**Figure 5:**
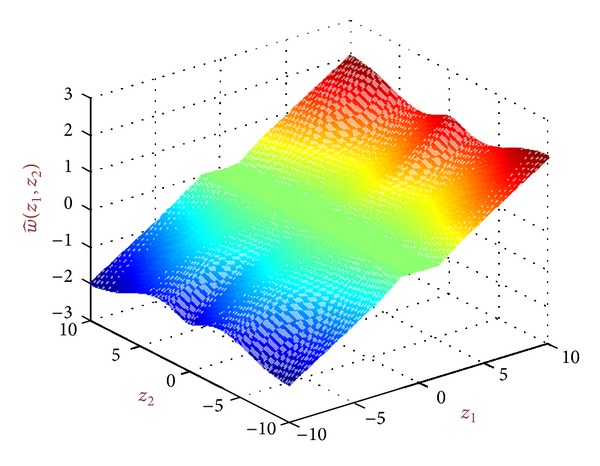
A shrinkage function produced from BiGaussRayMixShrink for sample parameters.

We call this bivariate local shrinkage function as *BiGaussRayMixShrinkL*.  Figure 5 shows this shrinkage function with sample constant parameters.

For implementation of our denoising algorithm, we must estimate the parameters *σ*_*ij*_(*k*) for *i*, *j* = 1,2, and *a*(*k*) (that are for noise-free data) from noisy observation. For AWGN, the noisy observation would be a Gaussian mixture model with parameters *a*(*k*),$\sqrt{\sigma_{n}^{2}+\sigma_{ij}^{2}(k)}$ for *i*, *j* = 1,2. So, the following local EM algorithm is used to obtain the parameters.

*E-step*

(34) $$\begin{array}{c} r_{1}\left(k\right)⟵\frac{a\left(k\right)g_{1}\left(\overset{-}{y}\left(k\right)\right)}{a\left(k\right)g_{1}\left(\overset{-}{y}\left(k\right)\right)+\left(1-a\left(k\right)\right)g_{2}\left(\overset{-}{y}\left(k\right)\right)},\\ r_{2}\left(k\right)⟵1-r_{1}\left(k\right). \end{array}$$

*M-step*

(35) $$\begin{array}{c} a\left(k\right)⟵\frac{1}{M}\underset{j\in N\left(K\right)}{\sum }r_{1}\left(j\right), \end{array}$$

(36) $$\begin{array}{c} \sigma_{1m}^{2}\left(k\right)⟵\frac{\sum_{j\in N\left(K\right)}^{}r_{i}\left(j\right)y^{2}\left(k\right)}{\sum_{j\in N\left(K\right)}^{}r_{i}\left(j\right)\;}-\sigma_{n}^{2},\;m=1,2, \end{array}$$

(37) $$\begin{array}{c} \sigma_{2m}^{2}\left(k\right)⟵\frac{\sum_{j\in N\left(K\right)}^{}r_{i}\left(j\right)y_{p}^{2}\left(k\right)}{\sum_{j\in N\left(K\right)}^{}r_{i}\left(j\right)}-\sigma_{n}^{2},\;m=1,2, \end{array}$$

where *M* is the number of coefficients in the window *N*(*K*) centered at $\overset{-}{y}(k)$. As discussed in the literatures [40], for non-Gaussian mixture models, which is a case for two-sided Rayleigh noise, using (34)–(36) finally converge to the final results. 

 Our denoising algorithm is summarized in Algorithm 1.

## 4. Shape Adaptive Windows Selection

It has been shown that using anisotropic and shape adaptive window for local parameter estimation can extremely improve the modeling and processing results. For example, in [47] a new image denoising is introduced that proposes an anisotropic window around each pixel of image and obtains the denoised pixel just by using the located data in the window. Comparing with the denoising methods that are based on proposing isotropic window around each pixel (e.g., [23, 27, 34, 42–46]), the proposed method in [47] is able to segment the image to rather smoothed regions before denoising due to anisotropic window selection that leads to improvement of denoising results. As explained before, the mixture model parameters in each subbands are estimated locally using an isotropic window around each voxel. In this section at first we explain the structure of macular OCT then we introduce 3D “linear polynomial approximation-intersection confidence interval” (LPA-ICI) method for applying shape adaptive window selection around each voxel in 3D DCWT domain. So we will try the despeckling results in 3D DCWT domain by choosing an anisotropic window (instead of isotropic) for estimating the parameters of mixture model in each subband locally.

### 4.1. OCT Structure

To select the shape-adaptive window, we must take a look at the special structure of OCT data. In ophthalmology, the OCT data shows detailed images from within the retina. The automated analysis of OCT images can be used for the image-guided retinal therapy. Every year, many people become blind as a result of age-related macular degeneration (AMD) due to affecting the central retina where our central vision is perceived. The most sight-threatening form of AMD is called exudative or wet AMD. Choroidal neovascularization (CNV) is a common symptom of the degenerative maculopathy wet AMD. A wealth of powerful new treatments for CNV, especially anti-VEGF agents, have become available very recently to restore normal visual function. The risk of ocular adverse events, including the devastating intraocular infection, endophthalmitis, increases with repeated intravitreal treatment injections, and the effects of chronic treatment with anti-VEGF agents on the retina are unknown. Ideally a more cost-effective, patient-specific dosing strategy with the minimally necessary number of anti-VEGF injections is required. With all the promise, these novel treatments will only reach their full potential when objective and early indices of treatment response are developed. Prior to the introduction of retinal OCT imaging, clinical assessment of whether the preservation or restoration of visual function is successful, which indeed is the ultimate goal of treatment, could only be obtained by measuring visual function. Unfortunately, visual function lags structural response and is cumbersome and noisy, and its reproducibility is limited. Two-dimensional OCT imaging of the retina was introduced several years ago, and was rapidly adopted, among others, to qualitatively measure macular structure as an indicator of AMD treatment response and for guidance of retreatment in CNV recurrence. It is now becoming clear that these simplified structural measures though leading indicators of visual function are inadequate, as they are based on simplified interpretation of single transverse slices of the macula, some patients do not recover visual function even though their total macular thickness has become normally thin after treatment, and others paradoxically gain visual acuity while their macula is still thickened. 

True 3D spectral OCT imaging, that became available in 2007 is fast (1.5 s per volume scan), allows full 3D retinal coverage at a much higher resolution and offers improved imaging of subtle differences in retinal structure. In the recent years [65, 66], 3D analysis of 3D OCT as an improved measure of subtle macular structure has been proposed motivated by various hypotheses as follows: *A model of retinal response to initial anti-VEGF treatment for CNV, based on quantitative 3D OCT-derived measures, can predict the timing of retreatment.*

On this base, developing analysis methods and approaches for 3D spectral OCT image analysis in the presence of wet AMD pathology (Symptomatic Exudate Associated Derangements or SEADs, also known as AMD-related cysts, vessel leakages, etc.) and assessing their performance by comparison to expert analyses are of utmost interest. Another interesting subject is determining how well the quantitative SEAD- and layer-derived measures from 3D OCT predict the patient-specific outcome parameters in response to postinduction anti-VEGF treatment in patients with CNV in order to predict the timing of retreatment.

**Figure 6:**
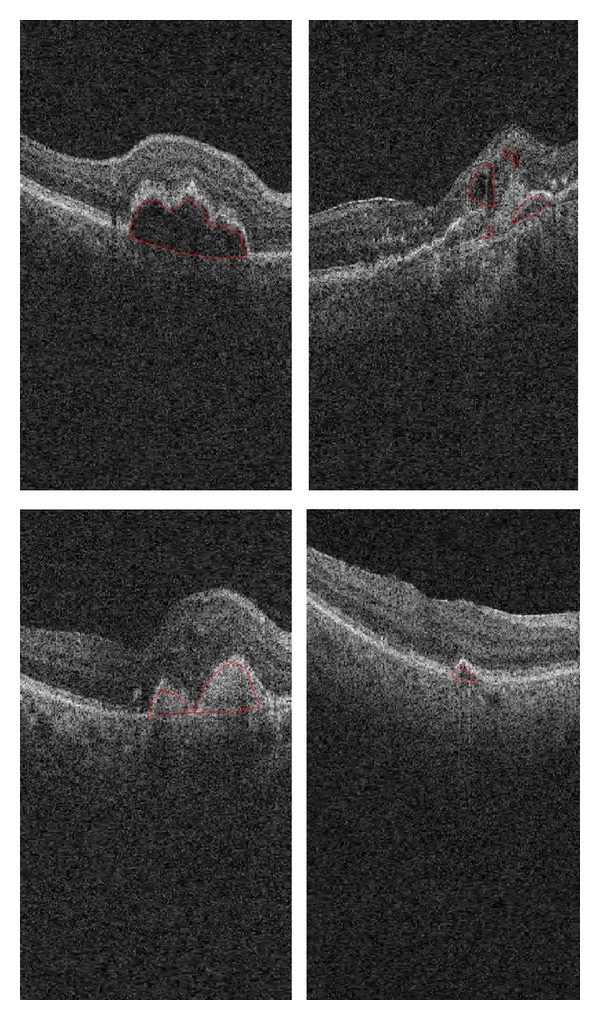
Macular OCTs and detected SEADs by an expert.

Figure 6 shows several sample macular OCTs and detected SEADs by an expert as the region of interest (ROI). As we can see in this figure, the most important information of OCT data (about retina layers) is located in the center of OCT images.

### 4.2. 3D LPA-ICI for Data between the First and Last Layers

In [47], a new image denoising based on using an anisotropic window around each pixel of image is introduced. To select the anisotropic window, the linear directional filters *g*_*h*,*θ*_ that are obtained using local polynomial approximation (LPA) are employed. The *θ* indicates the direction of filter that is a member of countable set {*θ*_1_, *θ*_2_,…, *θ*_*L*_}, where *L* is the number of directions. A common choice for *L* is *L* = 8 that results in the set {0°, 45°, 90°, 135°, 180°, 225°, 270°, 315°}. For each *θ*, the length of proposed window is selected from the countable and increasing set {*h*_1_, *h*_2_,…, *h*_*J*_}. So, for the noisy observation *y*(*k*), we would have the following estimate:

(38) $$\begin{array}{c} x_{h,\theta }^{\text{est}}\left(k\right)=g_{h,\theta }\left(k\right)*y\left(k\right). \end{array}$$

For each *θ* and *k*, an appropriate value of *h* called *h* ^+^ is estimated using the nonlinear intersection of confidence intervals (ICI) rule. *h* ^+^ is the largest *h* from the *h*_1_ < *h*_2_ < ⋯<*h*_*J*_ provided that the estimated data using *h* ^+^ does not have noticeable difference with the estimated data with smaller *h*'s. For this reason, the following confidence intervals are defined:

(39) $$\begin{array}{c} C_{s}=\left[x_{h_{s,\theta }}^{\text{est}}\left(k\right)-R\sigma_{x_{h_{s,\theta }}^{\text{est}}(k)},\;\;x_{h_{s,\theta }}^{\text{est}}\left(k\right)+R\sigma_{x_{h_{s,\theta }}^{\text{est}}(k)}\right], \end{array}$$

where *R* is the smoothing parameter and *σ*_*x*_*h*_*s*,*θ*__^est^(*k*)_ shows the variance of *x*_*h*_*s*,*θ*__^est^(*k*) and is obtained as follows:

(40) $$\begin{array}{c} \sigma_{x_{h_{s,\theta }}^{\text{est}}\left(k\right)}^{2}=\int P_{x_{h_{s,\theta }}^{\text{est}}(k)}\int \left(f\right)df=\int P_{y}\left(f\right)G_{h,\theta }\left(f\right)df, \end{array}$$

where *P*(·) shows the power spectral density function and *G*_*h*,*θ*_(*f*) is the Fourier transform of *g*_*h*,*θ*_, and for a white random process, (40) is simplified to

(41) $$\begin{array}{c} \sigma_{x_{h_{s,\theta }}^{\text{est}}\left(k\right)}^{2}=\int \sigma_{n}^{2}G_{h,\theta }\left(f\right)df=\sigma_{n}^{2}\sum g_{h,\theta }\left(k\right). \end{array}$$

According to the ICI rule, *D*_*s*_ is defined using the following formula:

(42) $$\begin{array}{c} D_{s}=\overset{s}{\underset{i=1}{⋂}}C_{i}. \end{array}$$

The largest *s* that leads to a nonempty value is called *s* ^+^, and finally *h* ^+^(*k*, *θ*) is obtained using *h* ^+^(*k*, *θ*) = *h*_*s*^+^_.

**Figure 7:**
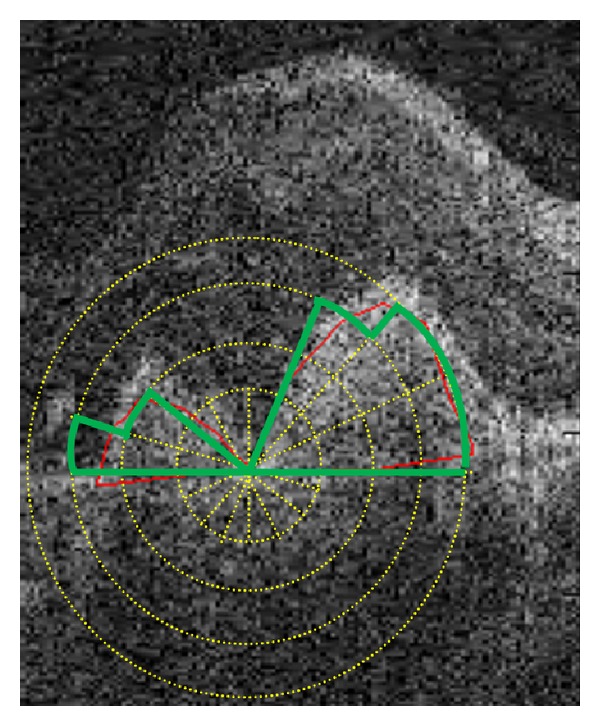
The red line shows the detected SEAD by an expert. The yellow circles show the isotropic windows with various radii. The green line illustrates the obtained anisotropic based on LPA-ICI rule.

Figure 7 shows an example of mentioned anisotropic window selection for a SEAD.

**Figure 8:**
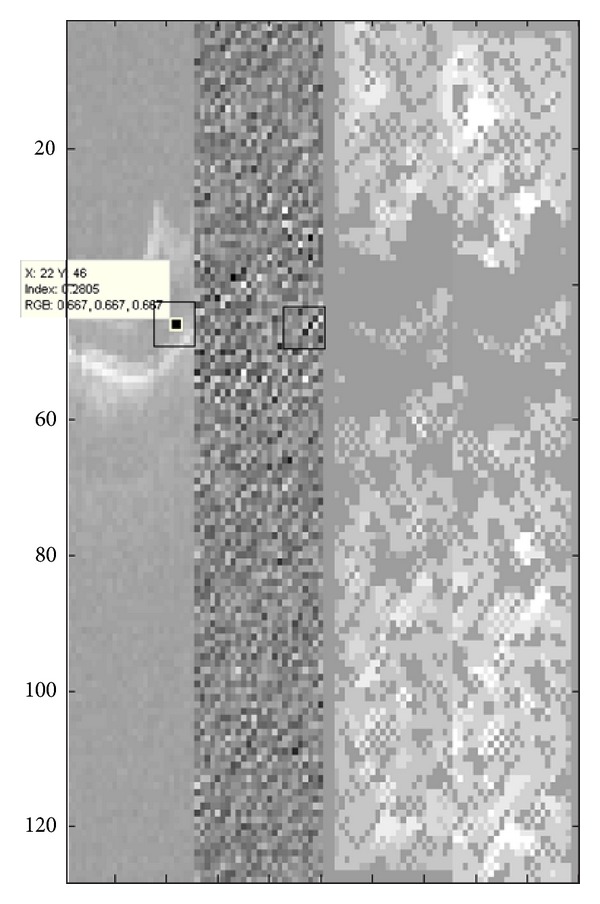
From left to right: imaginary LL subband of one slice of OCT data, the oriented (imaginary) subband around 45° (225°), *h* ^+^(·, 45°) for LL subband, and *h* ^+^(·, 225°) for LL subband extracted by applying LPA-ICI to the LL subband of imaginary part of DCWT. As indicated in the second image for *p*_1_ = (46,20), *p*_2_ = (47,21), and *p*_3_ = (46,22) we would have *h* ^+^(*p*_1_, 45°) = 2, *h* ^+^(*p*_1_, 225°) = 3 (green dash), *h* ^+^(*p*_2_, 45°) = 1, *h* ^+^(*p*_2_, 225°) = 3 (orange dash), and *h* ^+^(*p*_3_, 45°) = 3, *h* ^+^(*p*_3_, 225°) = 3 (red dash).

Since applying LPA-ICI in each subband is a time consuming process, a fast version of the mentioned algorithm can be based on only applying LPA-ICI to low-pass subbands using *L* = 12 with an offset of 15° that results in the set {15°, 45°, 75°, 105°, 135°, 165°, 195°, 225°, 255°, 285°, 315°, 345°} in a 2D case. Since, in this case, each subband is extracting the information concentrated in a specific direction corresponding to {15° (195°), 45° (225°), 75° (255°), 105° (285°), 135° (315°), 165° (345°)}, the extracted *h* ^+^(*k*, *θ*) = *h*_*s*^+^_ that results from applying LPA-ICI to corresponding low-pass subband is used for obtaining the local parameters of *k*th pixel. For example, suppose that DCWT is used for 3 scales and we want to calculate the local parameters of coarsest scale for the oriented real subband around 45° (225°). For this reason, *h* ^+^(*k*, 45°) and *h* ^+^(*k*, 225°) are extracted from the results of applying LPA-ICI on the LL subband of real part (or imaginary part) of DCWT. Then, if we are in the *j*th scale, only 2^*j*−1^ *h* ^+^(*k*, 45°) pixel in direction of 45° and 2^*j*−1^ *h* ^+^(*k*, 225°) pixel in direction of 225° are used to extract the local parameters of *k*th pixel in this subband (Figure 8).

**Figure 9:**
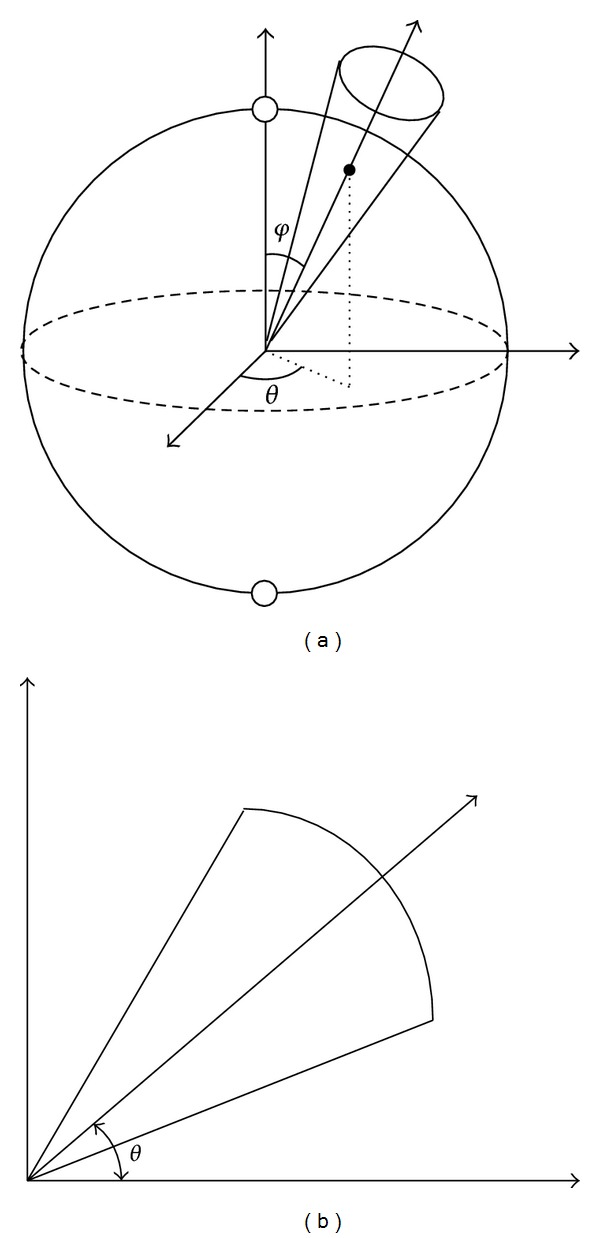
Comparison between a circular sector for direction *θ* in 2D case (b) with a conical body produced for direction (*θ*, *φ*) in 3D case (a).

A similar manner can be proposed in 3D case [67]. However, instead of using 2D direction *θ*_*i*_, we use 3D direction (*θ*_*i*_, *φ*_*i*_). As shown in Figure 9, in 2D case we use a circular sector for each direction while for 3D case a conical body is produced for direction (*θ*_*i*_, *φ*_*i*_), and the sphere is covered (partly) using these cones. Similar to 2D case *g*_*h*,*θ*,*φ*_, is defined and for each (*θ*_*i*_, *φ*_*i*_) the best *h* called *h* ^+^(*k*, *θ*_*i*_, *φ*_*i*_) is obtained using ICI rule.

Note that in order to incorporate the anisotropic window selection for each DCWT coefficient in our OCT denoising algorithm explained in Algorithm 1, instead of using a square window for parameter estimation, an anisotropic window is obtained for each coefficient *k* using the explained LPA-ICI method in this section, and only available data in this window are used for estimating *a*(*k*) and *σ*_11_(*k*),  *σ*_12_(*k*),  *σ*_21_(*k*), and *σ*_22_(*k*).

## 5. Experimental Results

**Figure 10:**
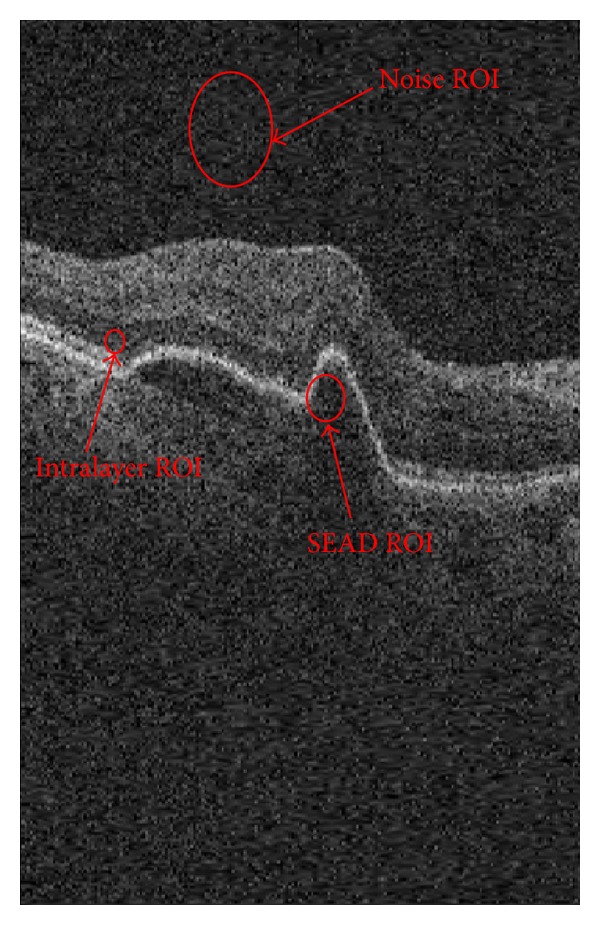
One slice from a sample OCT image and proposed ROIs for computation of MSNR and CNR reported in Table 1.

In this section, we apply the proposed despeckling algorithm to OCT image noise reduction. For this reason, we use 20 three-dimensional OCT datasets in the presence of wet AMD pathology (SEAD) and use mean signal-to-noise ratio (MSNR) and contrast-to-noise ratio (CNR) as two quality measurements for OCT data. To calculate these measurements, we must define the region of interest (ROI). In this paper, we propose this region within the SEAD as illustrated in Figure 10. The base MSNR and CNR are defined as follows:

(43) $$\begin{array}{c} \text{MSN}{\text{R}}_{\text{ROI}}=\frac{\mu_{\text{ROI}}}{\sigma },\\ \text{CNR}=\left|\text{MSN}{\text{R}}_{\text{ROI}1}-\text{MSN}{\text{R}}_{\text{ROI}2}\right|, \end{array}$$

where *μ*_ROI_ shows the mean of ROI and *σ* indicates the standard deviation of a large region outside the ROI (noise ROI in Figure 10).

**Figure 11:**
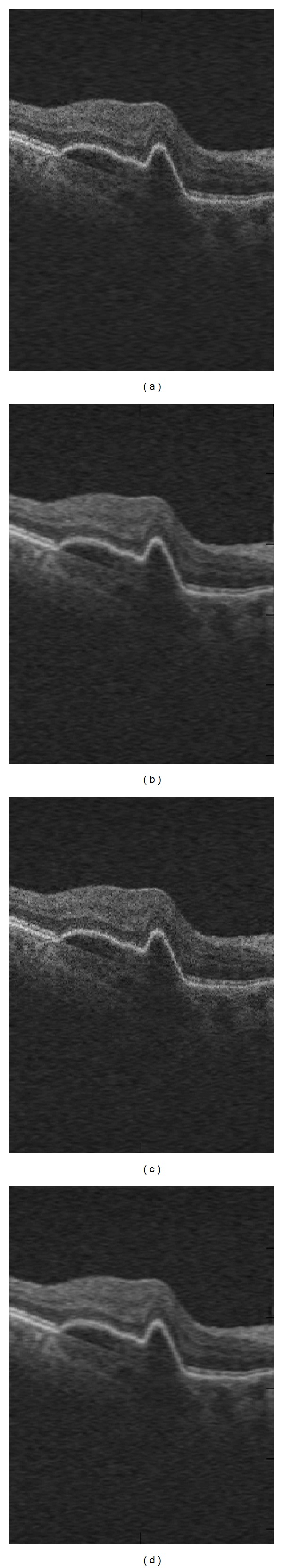
The results of applying homomorphic methods on proposed image in Figure 10. From top-left clockwise: despeckled data using *BiGaussMixShrinkL, *nonlocal* BiGaussMixShrinkL, *nonlocal* BiGaussRayMixShrinkL, *and *BiGaussRayMixShrinkL.*

**Figure 12:**
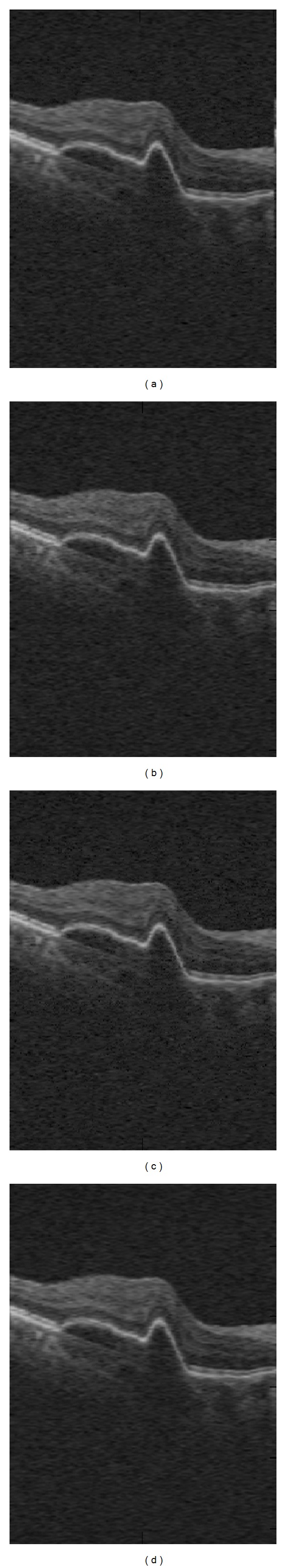
The results of applying non-homomorphic methods on proposed image in Figure 10. From top-left clockwise: despeckled data using *BiGaussMixShrinkL, *nonlocal* BiGaussMixShrinkL, *nonlocal* BiGaussRayMixShrinkL, *and *BiGaussRayMixShrinkL.*

**Figure 13:**
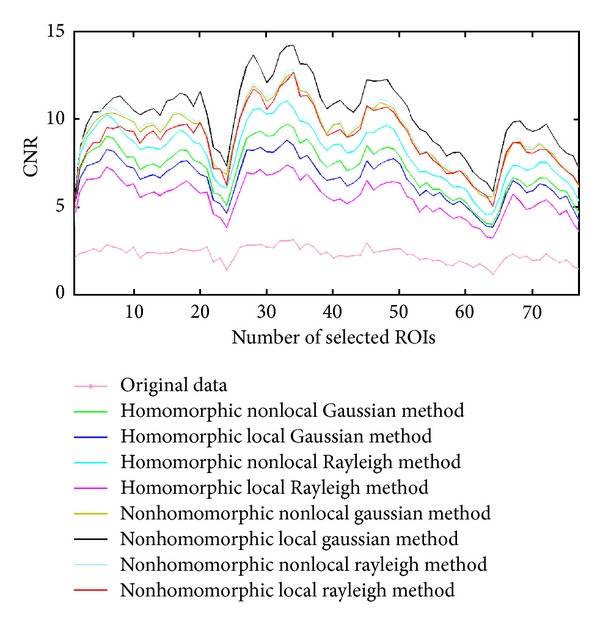
A comparison between CNR curves for 156 selected ROIs from OCT dataset.

Table 1 shows the results of MSNR and CNR for proposed ROIs in OCT data using our algorithm. As discussed in Section 3, various shrinkage functions can be obtained using our algorithm based on applying log transformation before applying 3D DCWT (we use homomorphic prefix for this method and non-homomorphic when we do not use log transformation) and proposing AWGN or two-sided Rayleigh pdf for modeling noise in 3D DCWT domain (we name them *BiGaussMixShrinkL *and* BiGaussRayMixShrinkL,* resp.). Figures 11 and 12, respectively, show the results of applying non-homomorphic and homomorphic methods for (a slice of) depicted OCT image in Figure 10. In this figure, also in Table 1, we compare the results of nonlocal version of methods to show the effect of using anisotropic window selection technique. In order to show the SNR improvements, CNR curves for 156 selected ROIs have been depicted in Figure 13. It is clear that non-homomorphic *BiGaussMixShrinkL *method outperforms the others.

**Figure 14:**
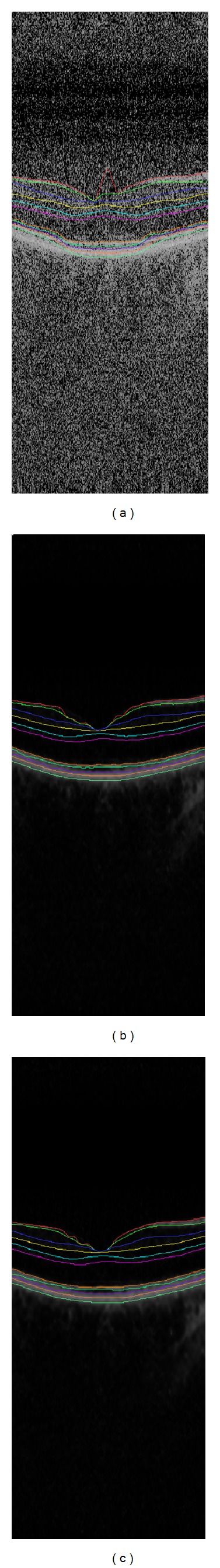
A comparison between the segmented layers of a 650 × 512 × 128 Topcon 3D OCT-1000 imaging system using proposed method in [53]. From left to right: original image, denoised image by nonlocal homomorphic *BiGaussRayMixShrinkL *method, and local homomorphic *BiGaussRayMixShrinkL *method.

Another way for evaluating the effect of our despeckling algorithm is the investigation of the intralayer segmentation results.  Figure 14 shows a comparison between the segmented layers of a 650 × 512 × 128 Topcon 3D OCT-1000 imaging system using proposed method in [53]. It is clear that the first layer is detected truly after despeckling.

## 6. Conclusion and Future Work

In this paper, we introduced a new noise reduction algorithm for 3D OCT data. We found new shrinkage functions employing a mixture of bivariate Gaussian for modeling wavelet coefficients in each subband of complex wavelets. The parameters of this mixture model are estimated locally using a shape-adaptive manner based on the special structure of OCT data. We also used this model for denoising of other kinds of noise. Experiments show that our model has better results than other methods visually and in terms of PSNR especially for the crowded images. In this paper, we suppose that the parameters of EM algorithm, in extracted windows are constant. It is possible to improve the EM algorithm, for example, by using recurrence equations. It is possible that we only propose the main section of data (between the first and last layers) containing retina layer information and apply our algorithm on the selected data to improve the speed and performance of denoising process.

Using 3D DCWT instead of other transforms such as 3D DWT is a main reason for improvement of the denoising results [30]. In [27], it has been shown that other kinds of oriented transforms such as steerable pyramid decomposition can produce better denoising results. However, for 3D case, 3D transforms that are applied on whole 3D data (not slice by slice) such as surfacelet [68] and 3D discrete curvelet [69] can be investigated.

## Figures and Tables

**Figure 15:**
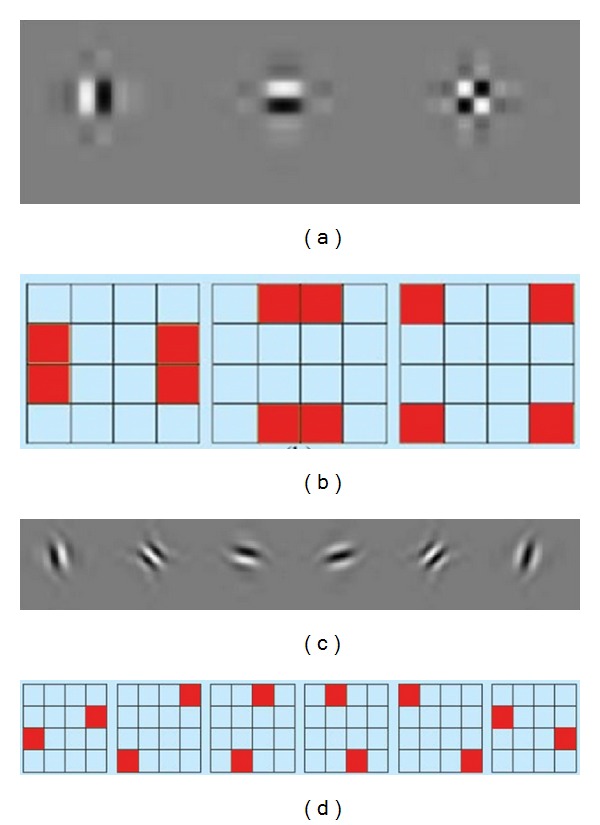
A comparison between subbands of DWT and DCWT. (a) The wavelets in the space domain (LH, HL, and HH). (b) The idealized support of the Fourier spectrum of each wavelet in the 2D frequency domain. We can see the checkerboard artifact of the third wavelet. (c) The complex wavelets in the space domain. (d) The idealized support of the Fourier spectrum of each wavelet in the 2D frequency plane. The absence of the checkerboard phenomenon is observed in both the space and frequency domains [48].

**Figure 16:**
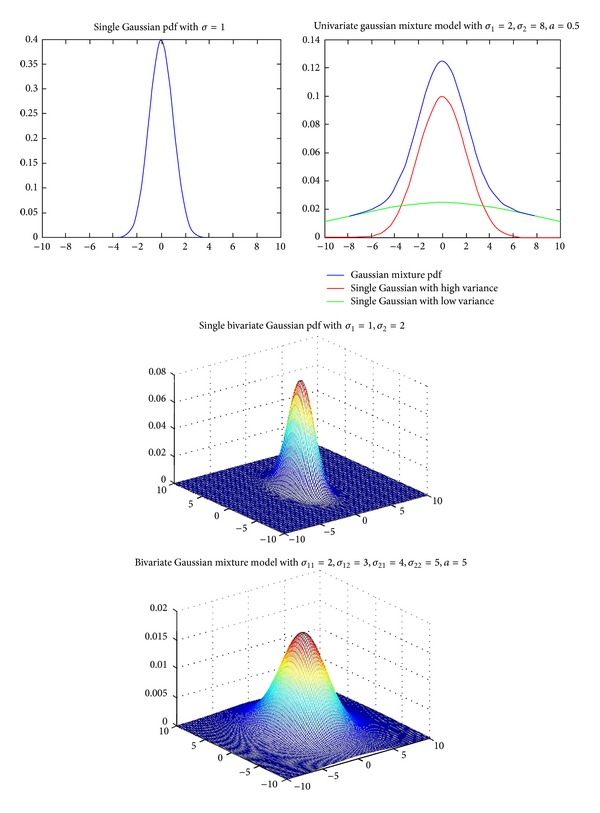
The pdf of a bivariate Gaussian mixture model for sample parameters and its marginal distribution.

**Figure 17:**
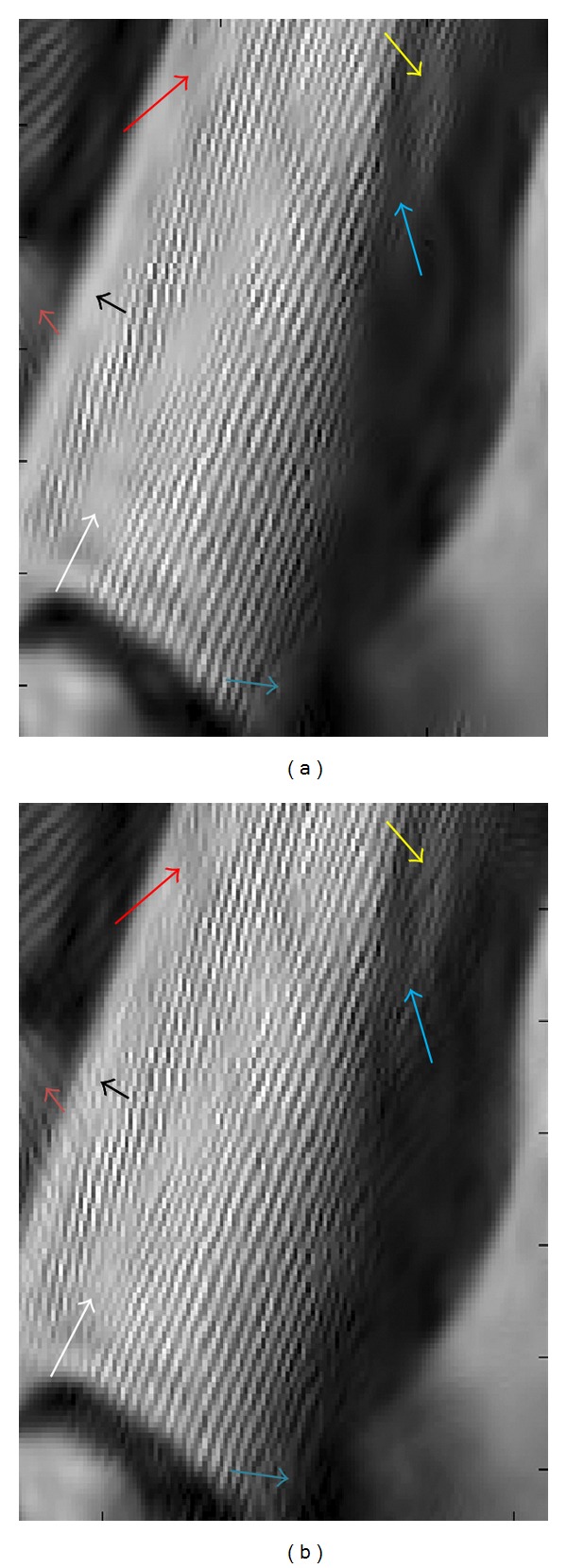
(a) shows a part of Barbara image denoised using BiShrink [45] for stationary noise with *σ*_*n*_ = 40 and (b) shows denoised image using our method. Comparing the area around the corresponding arrows, we understand that our method is able to better preserve the details of images.

**Figure 18:**
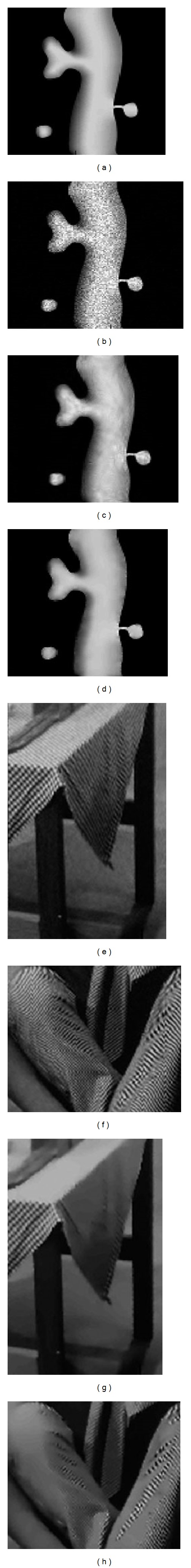
(a–d) show denoising results for Confocal Microscopy Phantom: from left to right: noise-free image, noisy image, and denoised image with our model and denoised image with Fast TI Haar algorithm. (e–h) show from left to right parts of denoised Barbara image with BiGaussMixShrinkL method and parts of denoised Barbara image with Fast TI Haar algorithm.

**Algorithm 1:**
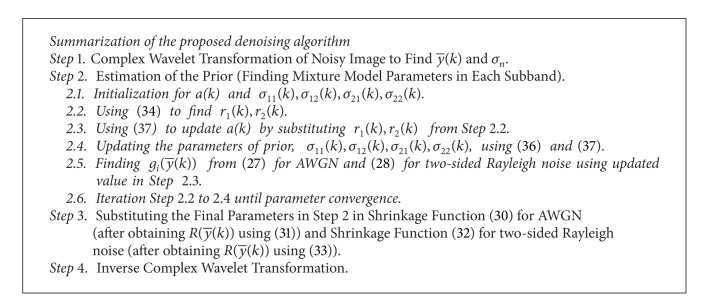
Outline of the proposed denoising algorithm.

**Table 1:** The results of MSNR and CNR using several ROIs, shown in Figure 12.

Methods			MSNR_ROI1_	MSNR_ROI2_	CNR
Local (L) Nonlocal (NL)	Homomorphic (H) Nonhomomorphic (NH)	Gaussian noise (G) Two-sided Rayleigh noise (R)			
L	H	G	7.00	15.76	8.76
NL	H	G	7.56	17.03	9.47
L	NH	G	12.27	27.76	13.49
NL	NH	G	10.77	22.73	11.95
L	H	R	5.89	13.11	7.22
NL	H	R	8.63	19.59	10.95
L	NH	R	10.75	22.55	11.81
NL	NH	R	10.88	23.05	12.17

	Original image		2.56	5.30	2.74

**Table 2:** PSNR (in dB) values of test images for different nonstationary noise levels.

Noise parameters σ_*g*_(*i*) = *k*_0_ *s*(*i*) + *k*_1_	Lena				Boat				Barbara			
	Noisy Image	Soft thresh. [54]	Proposed method in [55]	Our method	Noisy image	Soft thresh. [54]	Proposed method in [55]	Our method	Noisy image	Soft thresh. [54]	Proposed method in [55]	Our method
*k*_0_ = 0.05, *k*_1_ = 4	27.72	34.13	34.61	**35.60**	27.51	32.44	32.59	**33.26**	27.94	31.99	32.20	**33.56**
*k*_0_ = 0.1, *k*_1_ = 4	23.48	31.62	32.49	**33.31**	23.22	29.95	30.23	**30.75**	23.69	29.05	25.81	**30.91**
*k*_0_ = 0.2, *k*_1_ = 4	18.49	27.50	29.65	**30.52**	18.20	26.77	27.60	**28.13**	18.71	25.81	25.94	**27.91**

**Table 3:** Comparison between PSNRs (in dB) of denoised images with Fast TI Haar algorithm [55] and BiGaussMixShrinkL.

	Lena	Boat	Barbara	Confocal Phantom	Shep Logan Phantom	Bowl
Noisy image	27.22	27.05	27.49	35.74	47.68	28.21
Fast TI Haar	32.11	29.30	26.59	44.49	60.63	46.79
BiGaussMixShrinL	**39.88**	**37.57**	**37.78**	**47.36**	**64.65**	**47.09**

## Appendices

### A. Directional Selectivity Property of 2D DCWT

Since DWT in 2D domain is produced using separable (row-column) implementation, it has a poor directional selectivity. For example, the HH wavelet is the product of the high-pass functions along the first and second dimensions. Because DWT uses real filters, the HH wavelet mixes +45° and −45° orientations that results in the checkerboard artifact because it fails to isolate these orientations. In contrast, since the spectrum of the (approximately) analytic 1D wavelet is supported on only one side of the frequency axis, the spectrum of the DCWT in 2D domain is supported in only one quadrant of the 2D frequency plane.  Figure 15 illustrates a comparison between subbands of 2D DWT and 2D DCWT.

### B. A Sample Bivariate Gaussian Mixture Model

Figure 16 shows the pdf of a bivariate Gaussian mixture model for sample parameters. We can see the marginal distribution produced from the model in this figure.

### C. Other Kinds of Noise

In this appendix, we briefly explain the abilities of the proposed denoising algorithm in this paper for other kinds of noise.

#### C.1. Stationary Noise

We tested the shrinkage function *BiGaussMixShrinkL* for stationary noise and compared it with other methods such as ProbShrink [34], BiShrink [45], and BLS-GSM [27] and found that our algorithm outperforms these techniques visually and in terms of peak signal-to-noise ratio (PSNR) for various levels of noise. For example, our algorithm for crowded images preserves details of images while BiShrink [45] in some cases produces blurred images (e.g., compare the area around the corresponding arrows in Figure 17). In addition, in [40] using other bivariate mixture models such as bivariate Laplacian was examined. We compared the results of using our model with method in [40], and our simulations show that our algorithm is faster and outperforms the reported shrinkage functions in [40] for some images. For example, for a 512 × 512 8-bit grayscale Barbara image, an improvement of 0.6 dB is obtained for noise level of 30, and *BiGaussMixShrinkL* is two times faster.

#### C.2. Nonstationary Noise

This section presents nonstationary noise reduction examples in complex wavelet domain. Although the stationary noise model is able to simplify the implementation of denoising algorithms, the statistical properties of the noise are not always accurately described with this assumption. For example, in some applications, the noise statistics are spatially varying and the noise power varies between pixels or samples. In these cases, the nonstationary noise assumption is more reasonable and can improve the denoising results. For example, we contaminate three 512 × 512 grayscale images, namely, Lena, Boat, and Barbara using signal-dependent Gaussian noise with variance *σ*_*g*_(*i*) that is defined as a linear function of the pixel intensities *s*(*i*) as [54]:

(C.1) $$\begin{array}{c} \sigma_{g}\left(i\right)=k_{0}s\left(i\right)+k_{1}. \end{array}$$

Since the variance of each noise component is spatially varying with the corresponding content of signal, the nonstationary processes are able to model the statistical properties of this noise. A comparison between the denoised image using soft thresholding, proposed method in [54], and the denoised image using a mixture of two bivariate Gaussian pdfs with local parameters (*BiGaussMixShrinkL*) for different noise levels can be seen in Table 2. In this table, the highest PSNR value is bolded. We can see from the table that our proposed algorithm has the better results compared to others especially for the Barbara image (which contains details) in the high-level noise.

In [54], it has been shown that the proposed algorithm based on the signal-dependent Gaussian noise can also be effective for the reduction of Poisson noise. On this base, we use our algorithm for noise reduction of images corrupted by Poisson noise generated using corresponding voxel intensities. For this reason, we use the software provided on http://willett.ece.wisc.edu/software.html to compare our method with Fast TI Haar algorithm [55]. The PSNR of grayscale images Lena, Boat, Barbara, Confocal Microscopy Phantom, Bowl, and Shepp Logan Phantom can be seen in Table 3. We can see that our algorithm outperforms the Fast TI Haar algorithm.  Figure 18 illustrates a comparison between the denoised images produced from two algorithms. It is clear that our algorithm has better results especially for the crowded images. In fact, the Fast TI Haar algorithm has reasonable performance for soft images such as Confocal Microscopy Phantom, but since this algorithm blurs the produced images, for images with details such as Barbara, high-frequency features will be removed and we lose the important information.
